# Supplementary figures and images for: Integrating species and interactions into similarity metrics: a graph theory-based approach to understanding community similarity
Source: PeerJ. 2019 May 31;7:e7013. doi: 10.7717/peerj.7013 (PMC6546078; doi:10.7717/peerj.7013)

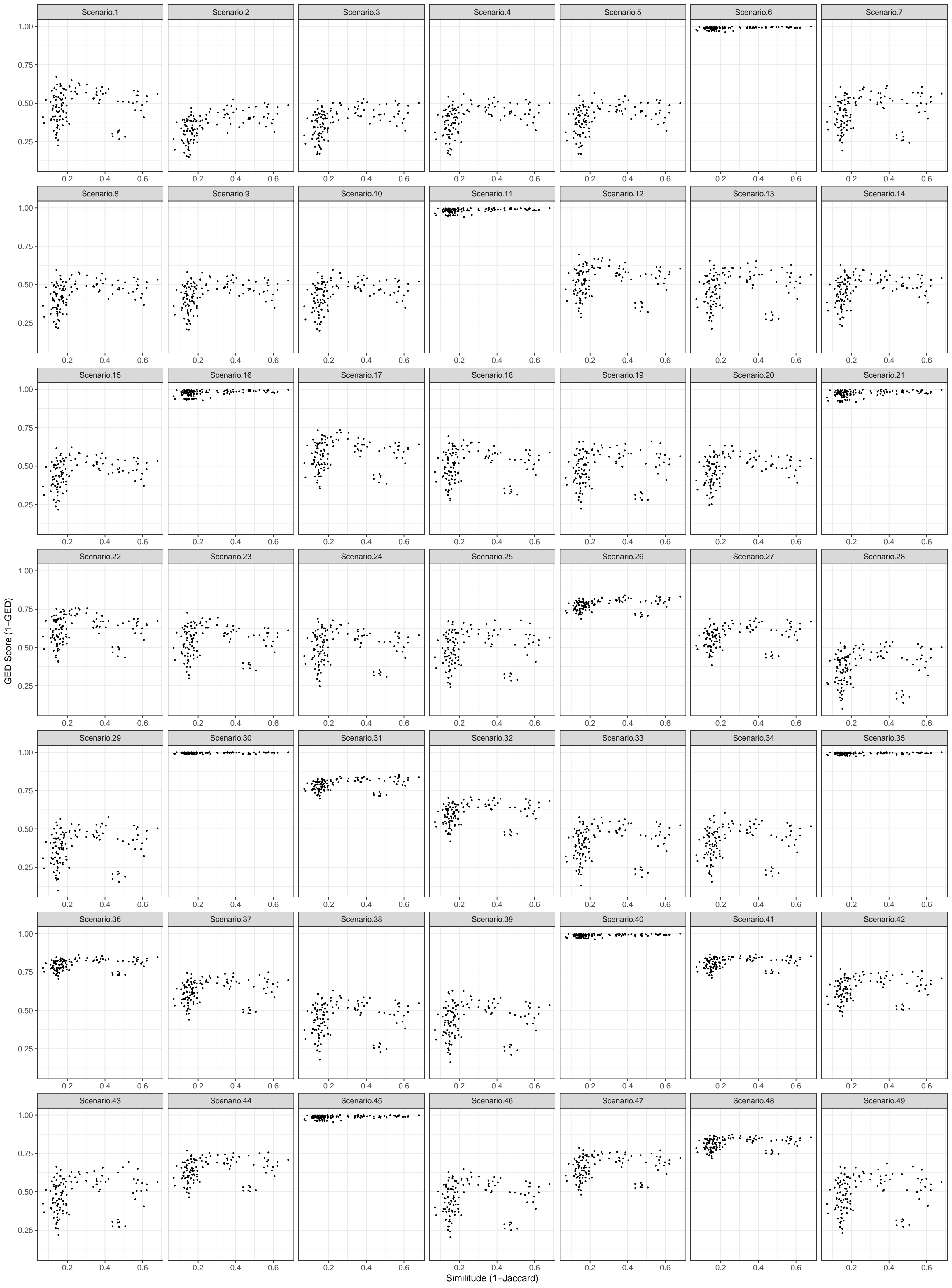

Supplement: Figure S1 — Forty-nine cost scenarios are included. Each point represents a pairwise comparison between food webs. [file peerj-07-7013-s001.pdf]
