# Supplementary material for: Integrating species and interactions into similarity metrics: a graph theory-based approach to understanding community similarity
Source: PeerJ. 2019 May 31;7:e7013. doi: 10.7717/peerj.7013 (PMC6546078; doi:10.7717/peerj.7013)
Supplement: Data S2 [file peerj-07-7013-s003.docx]

**Mutual information**

# where X and Y are two random variables

AMI<-function(X,Y,iter){

library(infotheo)

Vv<-rep(NA,iter)

Xv<-discretize(X)

Yv<-discretize(Y)

Mr<-mutinformation(Xv,Yv,method='mm')

EXv<-entropy(Xv,method='mm')

EYv<-entropy(Yv,method='mm')

MaxE<-max(c(EXv,EYv))

for(i in 1:iter){

Yvr<-sample(Yv$X)

Vv[i]<-mutinformation(Xv,Yvr,method='mm')

print(i)

}

MeanMI<-mean(Vv)

AMI<-(Mr-MeanMI)/(MaxE-MeanMI)

FIN<-matrix(c(EXv,EYv,Mr,MeanMI,AMI),nrow=1,ncol=5)

NN<-c('H(X)','H(Y)','MI','E(MI)','AMI')

colnames(FIN)<-NN

print(FIN)

}
